# Bundled assessment to replace on-road test on driving function in stroke patients: a binary classification model via random forest

**DOI:** 10.3389/fnagi.2025.1503672

**Published:** 2025-04-11

**Authors:** Lu Huang, Xin Liu, Jiang Yi, Yu-Wei Jiao, Tian-Qi Zhang, Guang-Yao Zhu, Shu-Yue Yu, Zhong-Liang Liu, Min Gao, Xiao-Qin Duan

**Affiliations:** ^1^School of Nursing, Jilin University, Changchun, China; ^2^Department of Rehabilitation, The Second Hospital of Jilin University, Changchun, China; ^3^School of Computer and Communication Engineering, University of Science and Technology Beijing, Beijing, China

**Keywords:** driving, stroke, eye-tracking, motor-cognitive functions, random forest

## Abstract

**Objectives:**

This study proposes to construct a model to replace the on-road test and provide a bundled assessment on the driving function of stroke patients.

**Methods:**

Clinical data were collected from 38 stroke patients who specified meeting criteria. Bundled assessment including the Oxford Cognitive Screen (OCS) scale ratings, eye tracking data obtained under the same eight simulated driving tasks as in subject 3, Fugl-Meyer Assessment-lower extremity (FMA-LE) scores, lower limb ankle muscle strength and active range of motion (AROM), and performance on the simulated driving machine. All patients were transported to a driving school and underwent the on-road test. The subject was classified as either Success or Unsuccess group according to whether they had completed the on-road test. A random forest algorithm was then applied to construct a binary classification model based on the data obtained from the two groups.

**Results:**

Compared to the Unsuccess group, the Success group had higher scores on the OCS scale for “crossing out the intact heart” (*p* = 0.015) and lower scores for “executive function” (*p* = 0.009). The analysis of eye tracking recordings revealed that the Success group exhibited a reduced pupil change rate, a higher proportion of eye movement types that were fixations, a longer mean fixation duration, and a significantly faster mean average velocity of saccade in the U-turn (*p* = 0.032), Left-turn (*p* = 0.015), and Free-driving tasks (*p* = 0.027). Compared to the Unsuccess group, the Success group had higher FMA-LE scores (*p* = 0.018), higher manual muscle strength for ankle dorsiflexion (*p* = 0.024) and plantarflexion (*p* = 0.040), and greater AROM in dorsiflexion (*p* = 0.020) and plantarflexion (*p* = 0.034). The success group demonstrated fewer collisions (*p* < 0.001), lane violations (*p* < 0.001), and incorrect maneuvers (*p* < 0.001) when completing the simulated driving task. The random forest model for bundled assessment demonstrated an accuracy of > 83% based on 56 statistically distinct input data sets.

**Conclusion:**

The bundled assessment, which includes cognitive, eye tracking, motor, and simulated driver performance, offers a potential indicator of whether stroke patients may be able to pass the on-road test. Furthermore, the established random forest classification model has demonstrated efficacy in predicting on-road test outcomes, which is worthy of further clinical application.

## 1 Introduction

Stroke is a prevalent disease that threatens the health and quality of life of middle-aged and elderly individuals (Cao and Elkins, 2024). In China, there are 1.5–2 million new stroke cases reported annually, with over 7 million survivors, 70% of whom experience varying degrees of disability (GBD 2019 Stroke Collaborators, 2021). Driving is an effective way for stroke survivors to participate in social activities (Collia et al., 2003; Akinwuntan et al., 2012) and is an integral part of daily life (Korner-Bitensky et al., 2006). On-road driving tests using a real car are the gold standard for evaluating driving ability (Devos et al., 2011). However, considering the high cost and inefficiency of on-road testing (Lee et al., 2003) and the lack of official guidelines in China, many stroke survivors would return to driving without on-road testing in conjunction with their clinician’s advice. Stroke survivors are at a greater risk of undergoing a motor vehicle crash than the average driver (Rabadi et al., 2010). Increased braking reaction time may result in a slower response to dynamically changing traffic conditions, thereby increasing the likelihood of driving accidents in stroke individuals (Montgomery et al., 2014). Multiple studies have sought to ascertain which cognitive metrics are predictive of driving performance in stroke patients by correlating cognitive scores with on-road performance (Ponsford et al., 2008; George and Crotty, 2010), simulator performance (Kotterba et al., 2005), or driving status (Perrier et al., 2010). The Trail Making Test (TMT) (Söderström et al., 2006), the Useful Field of View (UFOV) (Fisk et al., 2002; George and Crotty, 2010), and the Rey-O Complex Graphics Test (Akinwuntan et al., 2002; Akinwuntan et al., 2006) have been employed to assess driving ability in a diverse array of patient populations, including stroke survivors. However, the extant research findings have been inconclusive and contentious. The eye and pupillary response to cognitive processing provides valuable information for one’s higher cognitive function (Chen and Epps, 2013; Burley et al., 2017). Chan et al. (2023) found that compared to the cognitively impaired group, stroke patients in the group without cognitive impairment had higher eye saccade speeds, higher gaze path speeds, and shorter times to reach their goals. Eye tracking technology offers a valuable and reliable means of obtaining information that can be used to classify emotional and cognitive processes occurring during driving. Moreover, braking reaction time is dependent on motor and cognitive abilities (Lodha et al., 2021). Aslaksen et al. (2013) demonstrated that visuomotor information processing speed and motor dexterity were predictive of on-road driving performance in patients with traumatic brain injury and stroke. The strength and accuracy of ankle plantarflexion muscles influence braking time during driving in stroke survivors (Lodha et al., 2022). The current methods of assessing driving ability after stroke are primarily cognitive, motor, and simulator-based. However, the resulting conclusions are variable, and comprehensive ratings that combine all three are lacking.

The integration of motor and cognitive predictive on-road tests within the same driving simulator as the on-road test program would prove advantageous in terms of time and cost savings, while also offering a more beneficial experience for stroke patients. In this study, we employed the findings of the Chinese on-road test to identify bundled assessment including cognitive, motor, and simulator variables that influence the function of stroke patients to resume driving. A new driving assessment model based on the influencing factors was proposed using random forest as an alternative to the on-road test for a more scientific assessment of stroke patients’ suitability to return to driving.

## 2 Materials and methods

This is a cross-sectional study. The study was approved by the Ethics Committee of the Second Hospital of Jilin University (2023067). All patients who fulfilled the criteria and provided informed consent by completing an informed consent document were included in the study. We adhered to the STROBE reporting guidelines.

### 2.1 Participants

Thirty-eight patients with a diagnosis of stroke, aged between 18 and 70 years, were recruited for this study. All participants were inpatients in the rehabilitation medicine department of a tertiary hospital in Changchun, China. All participants had obtained a driving license and had previously driven independently. To facilitate effective communication with the subject during the assessment process, a Mini Mental State Examination (MMSE) score of between 24 and 30 is required (Tombaugh and McIntyre, 1992). Participants were excluded from the study if they met any of the following criteria: (1) with severe visual impairment, e.g., diplopia (2) with neurological or psychiatric disorders other than stroke, e.g., epilepsy (3) suffer from visuospatial neglect (fail in line bisection test).

### 2.2 Bundled assessment

All the participants were invited for an initial assessment to confirm that they met the inclusion criteria. Clinicians collected demographic information (including age, sex, etc.), clinical information (including stroke type, duration of illness, etc.), and driving-related information (including driving experience, preference for self-drive, etc.) from all participants on enrollment. Three experienced physiotherapists assessed motor, cognitive, and eye-tracking indicators in the simulated driving tasks, blind to the results of the driving test subgroup.

### 2.3 Cognition

The Oxford Cognitive Screen (OCS) is a simple, valid, and reliable tool for the assessment of cognitive deficits in patients with stroke [intraclass correlation coefficient (ICC) = 0.79–1.00] (Valera-Gran et al., 2019). It contains 10 subtests, providing 14 scores referring to five theoretically derived cognitive domains: attention, language, number, praxis, and memory (Demeyere et al., 2015). Lower scores on only one item of the OCS scale, “Executive task,” represent better functioning.

### 2.4 Eye tracking in a driving simulator

The trial was conducted in a quiet room with artificial lighting. A stationary eye-tracking device (Tobii Pro Fusion) was employed to record the participants’ eye-tracking at a sampling frequency of 250 Hz and an accuracy of 0.06° RMS. The top edge of the eye-tracking device was at the same level as the bottom edge of the screen. The distance between the screen and the participants ranged from 55 to 75 cm (TobiiProLab User Manual.pdf, n.d.). The data were collected and exported using Tobii Pro Lab (1.162). The driving simulator employed was AnLuDi (08), with a screen resolution of 1,920 × 1,080 and a refresh rate of 60 Hz. The driving software was Qiao Miao Xue Che, which was divided into seven sub-tasks (Starting, Lane Changing, Overtaking, Right Turn, U-turn, Left Turn, Parking) identical to the on-road test subject 3, as well as a section of Free-driving tasks. The first seven tasks required the patient to simulate routine driving maneuvers in an urban road environment, which included three motorways in each direction. The driving simulator uses a machine voice to remind the patient to complete each of the seven tasks in sequence and signals the completion of each task when finished. In the Free-driving task, patients were instructed to merge into traffic and drive toward the seaside, while also avoiding vehicles traveling in both the same and opposite directions. Additionally, they were required to manage road conditions such as traffic light changes and continuous curves on coastal mountain roads. We chose the entire screen as the Area of Interest (AOI). Several eye-tracking metrics were measured during the experiment, including general, fixation, and saccade. See Supplementary Table 1 for details of ET metrics definitions.

### 2.5 Motor function

The degree of motor impairments in the leg and foot was assessed using the Fugl−Meyer Assessment-lower extremity (FMA-LE) (Gladstone et al., 2002). Muscle strength in ankle dorsiflexion and plantarflexion was measured using the manual muscle test (MMT) (0, no muscle contraction; 5, normal strength) (Larsen, 2005). The maximal active range of motion (AROM) of dorsiflexion and plantarflexion of the ankle joint was quantified using a standard 7 inch, flat, clear plastic goniometer with 2° increments (ICC = 0.65–0.89) (Konor et al., 2012).

### 2.6 Classification of fitness to return to driving

All participants took part in the on-road test at the Junan Driving School in Changchun City on the day following the clinical evaluation. The test vehicle was an automatic car with dual brake control, and two driving school instructors were in the vehicle to ensure the safety of the participants. The participants drove the vehicle on the same route through the urban road sections around the driving school to complete the test, which was formally tested after a familiarization session with the route, with voice reminders from the testing deep throughout the test. The on-road test was divided into 15 sub-tasks, with specific judgment criteria referring to the grading rules of subject 3 in the “Motor Vehicle Driver Test Content and Methods.” The subject was classified as either Success or Unsuccess according to whether they had completed the on-road test. The two driving instructors decided whether the patient had passed the on-road test based on the entire route, with any disagreements being decided by the third driving instructor.

### 2.7 Classification using random forest

All basic statistical procedures were performed with the SPSS version 26.0. We used the one-sample Kolmogorov-Smirnov test to check the normality of the data distribution. The mean ± SD is used to express measures that obey a normal distribution, whereas those that do not obey a normal distribution are expressed as M (P 25–P 75). For the demographic and clinical data, the one-way analysis of variance (ANOVA) test or two independent samples non-parametric test was adopted in the group comparison between Success group and Unsuccess group. The level of statistical significance was set at *p* < 0.05. This study employed a random forest modeling approach to perform a binary classification task. The code is in Python language, the programming platform is the integrated development environment of PyCharm 2022. The random forest classifier function encapsulated in the scikit-learn library was directly utilized in the experiment. The successful group is set to the label “1” and the unsuccessful group is set to the label “2.” To reduce the risk of model overfitting, we restrict the depth of the trees and increase the minimum number of samples required for both leaf nodes and split nodes. The initial data is randomly divided into a training set and test set by “random_state,” and finally the model will output the accuracy and prediction of the test set. We performed multiple rounds of experimentation and parameter tuning, resulting in the following final model settings: n_estimators = 30, max_depth = 5, min_samples_leaf = 5, min_samples_split = 10, random_state = 42.

## 3 Results

### 3.1 Demographics and characteristics

This study recruited inpatients admitted to the hospital from November 2023 to June 2024. A cohort of 57 patients met the criteria after the initial assessment. Among them, 40 participants who were willing to return to driving were screened for evaluation. However, one individual withdrew due to oculomotor deficits during the nine-point calibration process, and another’s data was discarded due to his astigmatism, resulting in a low tracking ratio (< 50%). Thirty-eight participants ultimately completed all assessments and provided complete data for subsequent analyses. Of note, no adverse events were reported during the study. Of the 38 participants, 20 completed the on-road test (Success group), while the remaining 18 were in the Unsuccess group. The age range of the participants was 31–68, with the Success group being significantly younger than the Unsuccess group (*p* < 0.001). Males constituted a great majority of the subjects, and most were diagnosed with ischemic stroke. The characteristics of patients are presented in Table 1.

**TABLE 1:** Demographic characteristics of the participants.

Variable	Success (*n* = 20)	Unsuccess (*n* = 18)	*P*
**Demographic information**			
Age (y); mean (SD)	44.40 (9.05)	55.56 (9.65)	0.001**
Sex, male; *n* (%)	19 (95.00%)	17 (94.44%)	0.939
Years of education (y); mean (SD)	12.45 (2.44)	11.22 (2.92)	0.167
**Clinical information**			
Stroke type; ischemic/hemorrhagic	16/4	12/6	0.573
Duration of illness (m)	2.50 (1.00, 10.50)	1.50 (0.90, 3.50)	0.170
Handedness (right/left)	19/1	18/0	0.336
**Driving-related information**			
Driving experience (y)	15.00 (7.75, 20.00)	19.00 (6.75, 30.50)	0.529
Preference for self-drive (yes/no)	17/3	16/2	0.723
Driving hours per week (h); mean (SD)	11.93 (10.81)	11.67 (10.74)	0.942

**P < 0.01.

### 3.2 Comparison of cognitive abilities

The OCS scale scores for the Success and Unsuccess groups are presented in Table 2. Compared to the Unsuccess group, the Success group scored significantly higher on the “broken hearts” task (*p* = 0.015) and significantly lower on the “executive task” (*p* = 0.009). There were no between-group differences in the other OCS tasks although the Success group scored higher. Table 3 presents the eye-tracking metrics data for both groups for the eight simulated driving tasks. In all six simulated driving tasks (Starting, Lane Changing, Overtaking, Right Turn, U-turn, Left Turn) the Unsuccess group took longer to complete the task than the Success group, and in the Parking and Free Driving tasks, the Success group took longer to complete the task. Compared with Success group, the rate of pupil change was significantly larger in the Unsuccess group in all tasks. Except for the Parking task, the Success group had a significantly higher percentage of fixations in the eye movement type. Compared to the Unsuccess group, the mean duration of fixations was significantly longer in the Success group for all tasks, and the duration of first fixations was significantly longer in the Success group for the remaining tasks, except for the Free Driving task. In the Parking and Free Driving tasks, the Success group had a significantly longer total fixation time and a higher number of fixations than the Unsuccess group. In the other tasks, the Unsuccess group had a higher total fixation time and number of fixations but was only statistically different in the Right Turn task (*p* = 0.016). Compared to the Unsuccess group, the Success group had a faster velocity of saccade in all tasks but were only statistically different in the U-turn (*p* = 0.032), Left Turn (*p* = 0.015), and Free Driving (*p* = 0.027) tasks. There were no statistically significant differences between the two groups in the mean horizontal and vertical coordinates of the fixation point, the time of first fixation, the mean horizontal and vertical coordinates of the fixation point, and the total number of saccades across the eight tasks.

**TABLE 2:** Between-group differences in OCS scale items.

OCS scale items	Success group	Unsuccess group	*P*
Picture naming	4 (4,4)	4 (4,4)	0.238
Semantic	4 (4,4)	4 (4,4)	1.000
Orientation	4 (4,4)	4 (4,4)	0.131
Visual field	4 (4,4)	4 (4,4)	1.000
Sentence reading	4 (4,4)	4 (4,4)	0.131
Number writing	3 (3,3)	3 (2,3)	0.055
Calculation	4 (4,4)	4 (4,4)	0.890
Broken hearts	49.00 (47.00,50.00)	45.50 (36.75,49.25)	0.015*
Imitation	12 (12,12)	12 (11,12)	0.062
Verbal recall	4 (4,4)	4 (3,4)	0.099
Episodic recognition	4 (4,4)	4 (3.75,4)	0.122
Executive task	−1 (−1,−1)	−0.5 (−1,5.5)	0.009**

OCS, The Oxford Cognitive Screen. *P < 0.05, **P < 0.01.

**TABLE 3:** Between-group differences in eye tracking of eight driving simulation tasks.

Eye tracking metrics		Starting			Lane changing			Overtaking		
		**Success**	**Unsuccess**	***P***	**Success**	**Unsuccess**	***P***	**Success**	**Unsuccess**	***P***
General	Duration of interval	49.15 ± 14.13	60.65 ± 16.95	0.029*	44.01 ± 9.66	53.16 ± 12.29	0.015*	57.00 ± 9.49	67.43 ± 12.92	0.007**
	The pupil change rate	0.74 (0.39,1.53)	1.83 (0.58, 3.55)	0.026*	0.58 (0.24, 1.45)	0.97 (0.73, 3.41)	0.020*	0.68 (0.20, 1.52)	1.23 (0.68, 3.63)	0.012*
	Eye movement type	12.55 ± 5.28	7.81 ± 3.79	0.003**	12.08 ± 6.17	8.19 ± 4.51	0.034*	11.65 ± 5.30	8.10 ± 4.48	0.033*
	Gaze point X	1005.70 (975.43, 1045.70)	1011.11 (938.63, 1102.46)	0.599	979.47 (950.85, 1043.06)	1009.21 (953.03, 1096.35)	0.267	979.19 (948.58, 1028.57)	987.77 (940.18 ± 1061.22)	0.483
	Gaze point Y	674.89 ± 67.56	660.19 ± 61.39	0.489	653.72 ± 46.48	653.93 ± 58.00	0.990	651.53 ± 61.03	647.87 ± 66.45	0.861
Fixation	Total duration of fixations	28.22 (23.55, 32.65)	34.25 (20.04, 39.86)	0.483	27.74 (24.10, 34.84)	30.28 (17.89, 35.38)	0.861	39.89 ± 10.87	40.18 ± 15.68	0.947
	Average duration of fixations	388.35 ± 123.47	314.00 ± 69.81	0.031*	393.70 ± 103.42	311.83 ± 106.94	0.022*	347.00 (305.50, 496.25)	281.00 (245.50, 357.50)	0.039*
	Number of fixations	77.00 (50.50, 90.00)	93.50 (58.25, 128.00)	0.059	75.00 (65.00, 87.75)	92.00 (49.75, 111.50)	0.357	106.50 ± 38.63	134.28 ± 59.18	0.092
	Time to first fixation	151.00 (0, 655.00)	157.00 (15.75, 337.25)	0.496	108.00 (7.25, 774.00)	203.00 (0, 1259.00)	0.791	238.20 ± 179.16	303.50 ± 230.84	0.334
	Duration of first fixation	239.30 ± 153.41	116.94 ± 74.85	0.004**	263.25 ± 153.53	156.17 ± 119.09	0.023*	231.55 ± 124.88	133.67 ± 106.05	0.014*
	Fixation point X	1019.25 (988.51, 1044.12)	1009.94 (937.27, 1105.41)	0.884	981.22 (947.96, 1036.80)	1002.01 (922.60, 1072.99)	0.682	974.25 (954.70, 1022.00)	972.31 (891.25, 1062.25)	0.815
	Fixation point Y	669.46 ± 49.58	655.75 ± 61.88	0.454	646.11 ± 50.55	650.71 ± 62.97	0.805	645.70 ± 54.49	603.98 ± 163.72	0.289
Saccade	Number of saccades	52.95 ± 26.39	58.61 ± 29.89	0.539	51.45 ± 18.75	60.06 ± 26.39	0.251	78.70 ± 36.73	74.67 ± 39.24	0.745
	Average velocity of saccade	296.67 ± 108.21	235.83 ± 107.15	0.091	304.02 ± 102.15	256.08 ± 123.61	0.199	307.89 ± 109.44	270.75 ± 137.84	0.368
General	Duration of interval	80.60 ± 20.05	100.41 ± 24.29	0.009**	91.15 ± 14.70	101.87 ± 15.05	0.033*	104.46 ± 29.49	129.00 ± 36.96	0.029*
	The pupil change rate	0.64 (0.28, 0.89)	1.83 (0.46, 4.33)	0.025*	0.53 (0.22, 0.94)	3.31 (0.64, 4.96)	0.004**	0.64 (0.25, 1.38)	1.63 (0.89, 4.96)	0.003**
	Eye movement type	12.80 ± 5.21	8.47 ± 4.86	0.012*	10.39 ± 3.88	7.23 ± 3.83	0.016*	11.39 ± 4.25	8.16 ± 4.31	0.026*
	Gaze point X	1108.73 (1060.47, 1165.60)	1094.74 (1047.91, 1192.42)	0.930	833.90 (795.95, 868.50)	873.53 (768.14, 945.17)	0.279	847.81 (815.40, 887.52)	862.34 (824.32, 959.49)	0.169
	Gaze point Y	598.81 ± 41.54	585.82 ± 50.43	0.390	609.53 ± 49.19	596.91 ± 73.82	0.535	566.41 (554.55, 584.11)	569.82 (506.19, 601.33)	0.815
Fixation	Total duration of fixations	57.84 ± 19.35	67.00 ± 23.78	0.199	71.56 ± 19.69	65.10 ± 27.32	0.405	70.30 (63.35, 100.91)	91.56 (51.83, 110.81)	0.599
	Average duration of fixations	399.85 ± 176.25	303.39 ± 92.16	0.045*	359.50 ± 87.16	295.83 ± 94.20	0.037*	372.80 ± 88.20	304.78 ± 102.07	0.034*
	Number of fixations	158.00 ± 69.37	218.28 ± 77.97	0.016*	205.20 ± 64.65	216.78 ± 76.05	0.615	217.50 (159.75, 280.25)	269.50 (194.25, 382.00)	0.132
	Time to first fixation	340.00 (43.50, 2535.75)	194.50 (0.00, 1006.00)	0.332	267.00 (181.25, 800.75)	524.50 (133.75, 2383.00)	0.388	36.00 (0.00, 249.00)	178.00 (0.00, 999.50)	0.156
	Duration of first fixation	227.90 ± 117.63	150.78 ± 82.86	0.027*	234.75 ± 83.19	171.67 ± 81.29	0.024*	190.50 (145.50, 324.75)	138.00 (91.25, 193.50)	0.032*
	Fixation point X	1107.95 (1061.20, 1157.57)	1086.85 (1044.79, 1192.20)	0.861	835.69 (799.26, 869.47)	873.64 (771.44, 941.95)	0.306	846.89 (817.18, 883.00)	864.58 (827.59, 983.66)	0.161
	Fixation point Y	594.06 ± 37.62	577.74 ± 50.60	0.264	604.15 ± 48.10	590.85 ± 69.94	0.495	560.31 (550.25, 571.05)	557.49 (502.01, 590.18)	0.465
Saccade	Number of saccades	113.75 ± 63.87	126.33 ± 56.87	0.527	133.75 ± 56.13	155.56 ± 44.10	0.195	150.00 ± 65.97	173.11 ± 70.87	0.305
	Average velocity of saccade	339.47 ± 155.24	283.15 ± 249.77	0.404	313.25 ± 127.12	227.29 ± 107.49	0.032*	333.97 ± 136.88	234.96 ± 96.32	0.015*
General	Duration of interval	62.65 ± 15.41	57.71 ± 22.75	0.434	135.88 ± 34.66	103.53 ± 44.02	0.016*			
	The pupil change rate	0.70 (0.25, 1.29)	1.98 (0.57, 4.00)	0.009**	0.87 (0.33, 1.71)	1.95 (0.98, 4.81)	0.009**			
	Eye movement type	9.16 ± 4.30	7.30 ± 2.70	0.122	10.71 ± 3.90	8.25 ± 3.33	0.044*			
	Gaze point X	1202.90 (1143.19, 1265.87)	1197.52 (1008.92, 1321.62)	0.682	1123.83 ± 237.18	1020.27 ± 142.80	0.117			
	Gaze point Y	670.28 (637.97, 692.06)	644.97 (606.25, 706.08)	0.365	582.33 ± 59.31	542.47 ± 104.17	0.151			
Fixation	Total duration of fixations	43.93 ± 15.08	27.66 ± 16.43	0.003**	102.60 ± 35.72	68.15 ± 46.06	0.014*			
	Average duration of fixations	344.80 ± 83.32	286.33 ± 70.52	0.026*	364.30 ± 125.27	284.67 ± 91.12	0.033*			
	Number of fixations	132.50 ± 45.16	94.28 ± 51.54	0.020*	291.05 ± 95.29	213.11 ± 115.63	0.029*			
	Time to first fixation	88.50 (0.00, 686.00)	273.00 (0.00, 1259.75)	0.381	578.00 (0.00, 1384.75)	812.00 (0.00, 3513.50)	0.614			
	Duration of first fixation	192.00 ± 107.18	127.78 ± 71.17	0.038*	236.60 ± 142.70	227.22 ± 207.89	0.871			
	Fixation point X	1148.08 (1199.42, 1265.49)	1195.30 (1005.17, 1325.67)	0.726	1109.37 ± 120.38	1040.13 ± 128.31	0.095			
	Fixation point Y	664.76 (635.30, 691.86)	643.24 (599.81, 736.24)	0.397	573.37 ± 55.20	570.74 ± 62.92	0.891			
Saccade	Number of saccades	94.85 ± 42.67	79.50 ± 68.87	0.409	203.60 ± 96.59	149.50 ± 99.09	0.097			
	Average velocity of saccade	326.44 ± 154.80	292.34 ± 104.75	0.437	269.05 ± 110.63	198.92 ± 70.23	0.027*			

*P < 0.05, **P < 0.01.

### 3.3 Comparison of motor abilities

Compared to the Unsuccess group, the Success group had higher scores on the FMA-LE (*p* = 0.018), greater muscle strength in ankle dorsiflexion (*p* = 0.024) and plantarflexion (*p* = 0.040), and greater AROM in ankle dorsiflexion (*p* = 0.020) and plantarflexion (*p* = 0.034). No statistically significant difference was observed in lower limb Brunnstrom staging between the two groups (Table 4).

**TABLE 4:** Between-group differences in motor and driver simulator performance.

Motor and driver simulator performance		Success group	Unsuccess group	*P*
Motor	FMA-LE	29.65 ± 2.21	28.11 ± 1.53	0.018*
	MMT in ankle dorsiflexion	7 (6, 7)	6 (5, 7)	0.024*
	MMT in ankle plantarflexion	7 (7, 7)	7 (6, 7)	0.040*
	AROM in ankle dorsiflexion	13.55 ± 3.79	10.50 ± 3.91	0.020*
	AROM in ankle plantarflexion	29.45 ± 5.27	25.72 ± 5.14	0.034*
	Brunnstorm	4 (4, 5)	4 (4, 4.25)	0.116
Driver simulator performance	Total collisions	0 (0, 0)	1 (0, 2.25)	< 0.001**
	Total lane violations	0 (0, 1)	2 (1, 3)	< 0.001**
	Total incorrect maneuvers	1 (1, 2)	3 (2, 5)	< 0.001**

FMA-LE, Fugl−Meyer Assessment-lower extremity; MMT, the manual muscle test; AROM, active range of motion.

*P < 0.05,

**P < 0.01.

### 3.4 Comparison of driver simulator performance

Compared to the Unsuccess group, the Success group performed better on the driving simulator, with significantly fewer total collisions (*p* < 0.001), total lane violations (*p* < 0.001), and total incorrect maneuvers (*p* < 0.001) (Table 4).

### 3.5 Accuracy of random forest

Table 5 lists 56 indicators with differences between groups as the input data of the random forest model. The set label is “1” for the Success group and “2” for the Unsuccess group. The experiment was divided into two rounds. In the early stage, the data from 38 patients were randomly divided into the training set and test set. To reduce the influence of data distribution on the results, several groups of repeated experiments were carried out in this experiment. In the three random classification results, the accuracy is 0.83 on all three times. A bar chart depicting the importance of the top 20 features in the random forest model is presented in Supplementary Figure 1. The top five features included: duration of interval in Line Changing, total collisions, duration of fixations in Stopping, total incorrect maneuvers, and AROM in ankle dorsiflexion.

**TABLE 5:** List of indices used in the random forest models.

Test	Indices	Test	Indices
Baseline	Age	ET of turning left	Duration of interval
OCS	Broken heart	–	The pupil change rate
	Executive task	–	Eye movement type
ET of starting	Duration of interval	–	Average duration of fixations
	The pupil change rate	–	Duration of first fixation
	Eye movement type	–	Average velocity of saccade
	Average duration of fixations	ET of stopping	Duration of fixations
	Duration of first fixation	–	The pupil change rate
ET of changing lanes	Duration of interval	–	Average duration of fixations
	The pupil change rate	–	Duration of first fixation
	Eye movement type	–	Number of fixations
	Average duration of fixations	ET of free driving	Duration of interval
	Duration of first fixation	–	The pupil change rate
ET of overtaking	Duration of interval	–	Eye movement type
	The pupil change rate	–	Average duration of fixations
	Eye movement type	–	Duration of fixations
	Average duration of fixations	–	Number of fixations
	Duration of first fixation	–	Average velocity of saccade
ET of turning right	Duration of interval	Motor	FMA-LE
	The pupil change rate	–	MMT in ankle dorsiflexion
	Eye movement type	–	MMT in ankle plantarflexion
	Average duration of fixations	–	AROM in ankle dorsiflexion
	Duration of first fixation	–	AROM in ankle plantarflexion
	Number of fixations	Driver simulator performance	Total collisions
ET of making a U-turn	Duration of interval	–	Total lane violations
	The pupil change rate	–	Total incorrect maneuvers
	Eye movement type	–	–
	Average duration of fixations	–	–
	Duration of first fixation	–	–
	Average velocity of saccade	–	–

OCS, The Oxford Cognitive Screen; ET, eye tracking; FMA-LE, Fugl−Meyer Assessment-lower extremity; MMT, the manual muscle test; AROM, active range of motion.

The data from 38 patients were randomly divided into the training set and test set:

random_state = 10


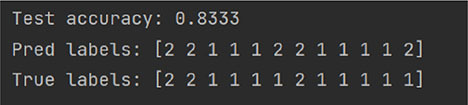


random_state = 42


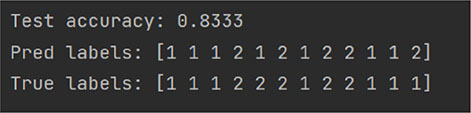


random_state = 75


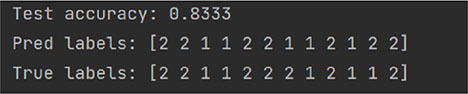


We randomly selected 12 patients as the validation set using a 7:3 split ratio (Random_state = 42) to evaluate the model’s performance based on the confusion matrix. The model correctly predicted six individuals of label “1” and four individuals of label “2.” As a result, the model achieved a sensitivity of 80.00%, a specificity of 85.71%, a false-positive rate of 14.29%, and a false-negative rate of 20.00%.


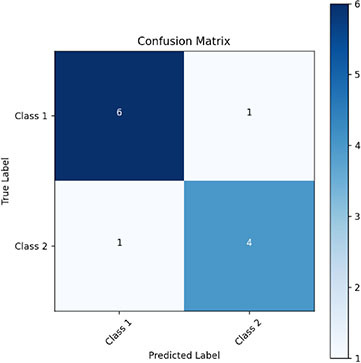


## 4 Discussion

### 4.1 Characteristics of driving ability in stroke patients between groups

Stroke can lead to a variety of dysfunctional consequences that can affect driving behaviors. In this study, the analysis of the Success and Unsuccess groups based on on-road test results revealed differences in bundled assessment between the two groups to varying degrees. The results of the baseline data indicated that the Success group was younger, suggesting that younger stroke patients were more likely to resume driving. The results of the between-group differences on the OCS showed that the Success group was more attentive, with better organization of instructions, switching, and decision-making. The lack of differences on the other OCS items may be because patients other than those with mild cognitive dysfunction had already been excluded from the criterion using the MMSE. This finding is consistent with previous research indicating a positive correlation between cognitive scale scores and driving scores (Bekiaris et al., 2003).

The results of the eye-tracking metrics of fixation also corroborate this finding. The Success group demonstrated a diminished pupil change rate and a heightened percentage of fixation in the eye-movement type across all tasks. This suggests that the Success group had a more straightforward visual search and target localization for key operations in the driving task, with less cognitive effort. Consequently, they were able to identify and focus on useful information, such as road turning points and turn signals, more expediently without frequent saccades. The longer mean fixation duration in the Success group indicates that they employed a deliberate visual search strategy, aiming to gather as much information as possible at each fixation point and to maintain focus on a specific visual element. The Unsuccess group is more prone to distraction problems, which may affect the ability to fully perceive and react to the surroundings while driving. These outcomes are in alignment with prior research indicating a positive correlation between fixation duration and attention, as well as working memory (Skaramagkas et al., 2023), and that longer periods of fixation are associated with better cognitive outcomes (Yantis et al., 2012). Except for the free-driving task, the first fixation duration was longer for the Success group. This may be because the Free Driving task was more complex, and the Success group needed to spend less fixation time to double-check the safety of their surroundings, thus reducing the between-group differences. The Parking task required the vehicle to be within 30 cm of the road edge line, while the Success group took longer to adjust. Since the Success group did not trigger the abort task due to the low number of collisions in the Free-driving task, only these two tasks took significantly longer to complete. Accordingly, the longer the duration of all completed tasks, the more the total time and number of fixations. However, only in the Right turn task, the Unsuccess group looked significantly more often than the Success group, which may be partly due to the reduction in single fixation time and partly related to the reduced orientation and spatial cognition in the Unsuccess group. Similar to pupil dilation, saccades reflect the information processing capacity of stroke patients, which is also influenced from above by higher-level attentional processes (Hess and Polt, 1960). The Success group had a faster velocity of saccade in all tasks, suggesting that the Success group could efficiently and quickly find targets or information of interest. However, statistical differences were observed only in the U-turn, Left turn, and Free-driving tasks, suggesting that these three tasks were more challenging and the Unsuccess group’s velocity of saccade was significantly slower. Similarly, Siegenthaler et al. (2014) showed that the velocity of saccade decreases as task difficulty increases. One potential explanation is that elevated working memory loads result in heightened information processing demands, which in turn lead to fixation difficulties and a delay in the saccade to the subsequent position (Krejtz et al., 2018). Considering the homogeneity of the task, no statistically significant difference was observed between the two groups in terms of fixation point, etc. This may be due to the absence of a notable discrepancy between the two groups in terms of their pre-morbid driving experience and proficiency. It can be inferred that the observed variations in driving performance are more likely to be attributable to the presence of disparate cognitive and motor dysfunctions after stroke.

Driving requires precise foot motion to control the accelerator and brakes (Kim et al., 2019). Ankle mobility and muscle strength were better in the Success group, suggesting that these patients were more flexible and stable when controlling the accelerator and brake. These findings indicate that strengthening lower limb function, particularly the ankle joint, maybe a pivotal aspect of restoring driving ability in post-stroke rehabilitation training (Lodha et al., 2016). However, no statistically significant difference was observed in Brunnstrom’s staging between the two groups. On the one hand, this may be because the Brunnstrom staging is relatively crude and does not allow for a precise assessment of the differences in lower limb function, and on the other hand, it is not a direct reflection of the patient’s performance in the driving task.

### 4.2 Modeling evaluation

The data of 38 patients collected in the early stage were randomly distributed according to the training set: test set = 7:3. In many experiments, it was observed that the accuracy rate was high, and the average accuracy rate was over 83%. The data experiments show that the model can effectively replace the on-road test to complete the safe and low-cost assessment of the driving ability of stroke patients, achieving the same effect as the on-road test. However, this model is a binary classification model, which is only suitable for distinguishing whether a patient is suitable for returning to driving. Although we identified features that influence the random forest model, they did not account for a high proportion of importance. Therefore, the results should be interpreted with caution. Among them, total collisions and incorrect maneuvers directly reflect driving performance. Additionally, the findings suggest that attention and ankle dorsiflexion mobility are relatively important in determining the suitability of stroke patients for return to driving. Our model demonstrates high specificity (85.71%), effectively minimizing the risk of incorrectly identifying stroke patients who are unfit to drive as eligible for return to driving. While the sensitivity of 80.00% is not perfect, it still provides reasonable assurance that suitable patients can be correctly identified. The false positive rate of 14.29% indicates that the model performs well in excluding unfit drivers, contributing to overall safety. However, the potential safety risks associated with false positives should not be overlooked. Future improvements can be made by expanding the sample size, optimizing feature selection, and exploring alternative algorithms to further enhance model performance.

### 4.3 Clinical implications

To gain a deeper understanding of the specific influencing factors of return to driving, the bundled assessment considered four aspects: cognitive, eye tracking, motor, and simulated driver’s performance. Our study proactively investigated alternative assessment techniques to the costly and hazardous on-road test. It streamlined the assessment, reduced the assessment duration, and furnished theoretical backing for clinicians to provide clinical counsel about returning to driving. This study is the initial investigation into bundled assessment on the driving functions of stroke patients in conjunction with the content of the Chinese driving test, with the objective of facilitating patients’ return to their careers and lives.

### 4.4 Limitations

The study has some limitations. Firstly, we did not consider potential confounders due to the disease heterogeneity of stroke, such as lesion volume and region of involvement. These factors could affect the interpretation of the results. Secondly, the external validity and generalizability of the findings are uncertain as the study was conducted in a single center in China, with a small sample (*n* = 38). Different driving rules, licensing policies and road conditions in foreign countries may lead to different results. Further confirmation from multi-center, large sample size pilot studies is still needed in the future. Finally, although this study is designed to simulate a real driving environment, it is constrained by the availability of instrumentation and the inability to account for the potential influence of emotional changes on eye-tracking indexes during driving. The next step will involve further refinement of the model through the incorporation of immersive simulated driving scenarios.

## 5 Conclusion

The bundled assessment, which including cognitive, eye tracking, motor and simulated driver performance, offers a potential indicator of whether stroke patients may be able to pass the on-road test. Furthermore, the established random forest classification model has the potential to simplify the assessment items and replace the on-road test, thereby enabling an objective assessment of whether stroke patients are suitable for safe driving.

## Data Availability

The original contributions presented in this study are included in this article/Supplementary material, further inquiries can be directed to the corresponding author.
