# Supplementary material for: Bundled assessment to replace on-road test on driving function in stroke patients: a binary classification model via random forest
Source: Front Aging Neurosci. 2025 Apr 11;17:1503672. doi: 10.3389/fnagi.2025.1503672 (PMC12021917; doi:10.3389/fnagi.2025.1503672)
Supplement: Supplementary file 1 [file Data_Sheet_1.docx]

Supplementary Table 1. Details of eye tracking metrics definitions.

| Metric name | Description | Unit |
| --- | --- | --- |
| General | | |
| Duration of  interval | The duration of an interval. | Seconds |
| The pupil change rate | Refers to the ratio of the amount of change in pupil diameter to the initial pupil diameter. | % |
| Eye movement  type | Ratio of the number of times identified as fixation to the number of times identified as saccade when eye movement data are acquired and recognized at 250 Hz. | / |
| Gaze point X | This is the average x-coordinate of each sampled gaze point. | Pixels  (DACS) |
| Gaze point Y | This is the average y-coordinate of each sampled gaze point. | Pixels (DACS) |
| Fixation | | |
| Total duration of fixations | The total duration of the fixations inside this area of interest during an interval. | Milliseconds |
| Average duration of fixations | The average duration of the fixations inside this area of interest during an interval. | Milliseconds |
| Number of  fixations | The number of fixations occurring in this area of interest during an interval. | Count |
| Time to first  fixation | The time to the first fixation inside this area of interest during an interval. | Milliseconds |
| Duration of first fixation | The duration of the first fixation inside this area of interest during an interval. | Milliseconds |
| Fixation point X | This is the average x-coordinate of the fixation point. | Pixels (DACS) |
| Fixation point Y | This is the average y-coordinate of the fixation point. | Pixels (DACS) |
| Saccade | | |
| Number of  saccades | The number of saccades occurring during an  interval. | Count |
| Average velocity of saccade | The average velocity of saccade in this area of interest during an interval. | Degrees/second |

DACS=display area coordinate system

Supplementary Figure 1. Features Importance In Random Forests.


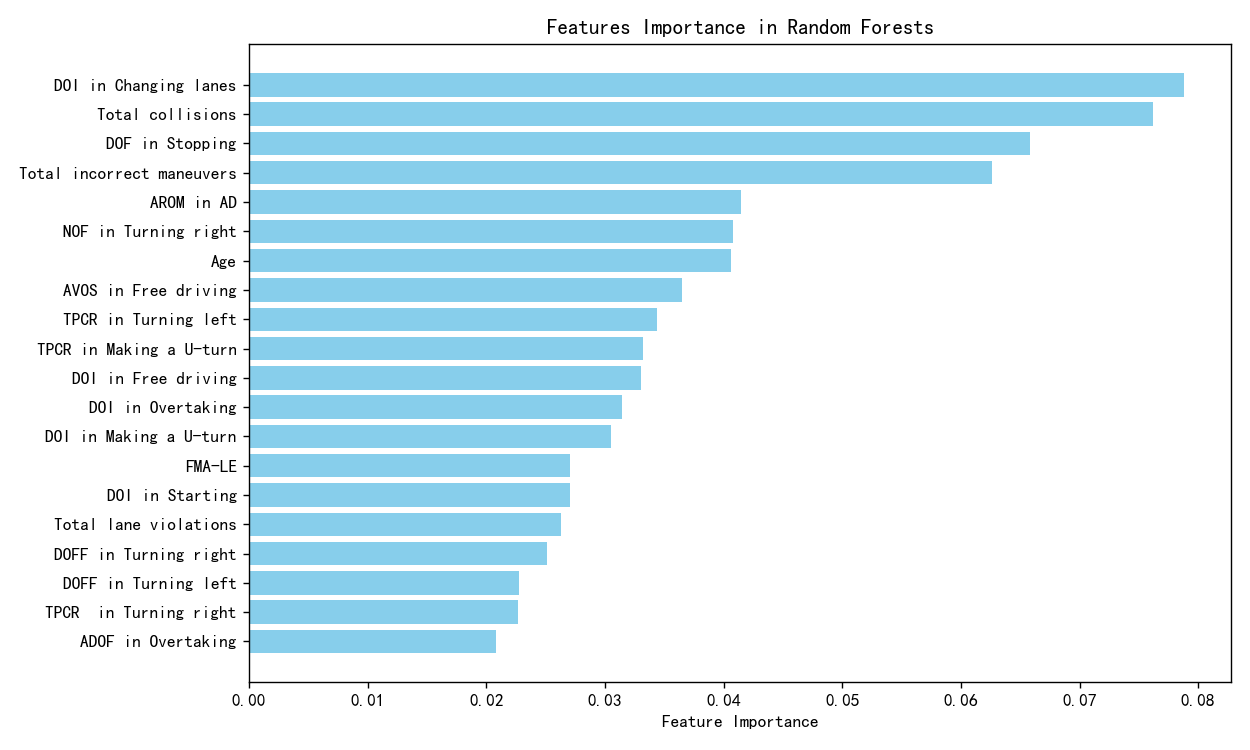


DOI= Duration of interval; DOF= Duration of fixations; AROM=active range of motion; AD= ankle dorsiflexion; NOF= Number of fixations; AVOS= Average velocity of saccade ; TPCR= The pupil change rate; FMA-LE= Fugl−Meyer Assessment-lower extremity ; DOFF= Duration of first fixation; ADOF= Average duration of fixations.
